# Nonalcoholic and Alcoholic Beverage Intakes by Adults across 5 Upper-Middle- and High-Income Countries

**DOI:** 10.1093/jn/nxaa324

**Published:** 2020-11-26

**Authors:** Lana Vanderlee, Christine M White, Sharon I Kirkpatrick, Vicki L Rynard, Alejandra Jáuregui, Jean Adams, Gary Sacks, David Hammond

**Affiliations:** NUTRISS Centre, School of Nutrition, Laval University, Québec, Québec, Canada; School of Public Health and Health Systems, University of Waterloo, Waterloo, Ontario, Canada; School of Public Health and Health Systems, University of Waterloo, Waterloo, Ontario, Canada; School of Public Health and Health Systems, University of Waterloo, Waterloo, Ontario, Canada; School of Public Health and Health Systems, University of Waterloo, Waterloo, Ontario, Canada; Centre for Health and Nutrition Research, National Institute of Public Health, Cuernavaca, Mexico; Centre for Diet & Activity Research, Medical Research Council Epidemiology Unit, University of Cambridge, Cambridge, United Kingdom; Global Obesity Centre, Deakin University, Geelong, Victoria, Australia; School of Public Health and Health Systems, University of Waterloo, Waterloo, Ontario, Canada

**Keywords:** sugar-sweetened beverages, sugary drinks, alcohol, diet beverage consumption, beverage consumption, noncaloric sweetener, international comparisons, adults

## Abstract

**Background:**

Despite considerable public health interest in sugary drink consumption, there has been little comparison of intake across countries.

**Objectives:**

This study aimed to compare the consumption frequency and amounts of commonly consumed beverages among adults in 5 upper-middle- and high-income countries, and examine differences in consumption between population subgroups.

**Methods:**

Adults aged 18–65 y completed online surveys in December 2017 in Australia (*n* = 3264), Canada (*n* = 2745), Mexico (*n* = 3152), the United Kingdom (*n =* 3221), and the USA (*n* = 4015) as part of the International Food Policy Study. The frequency of consuming beverages from 22 categories in the past 7 d was estimated using the Beverage Frequency Questionnaire. Regression models were used to examine differences in the likelihood of any consumption and in the amounts consumed of sugar-sweetened beverages (SSBs), sugary drinks (SSBs and 100% juice), diet, and alcoholic beverages between countries and across sociodemographic subgroups.

**Results:**

The prevalence of reported SSB consumption in the past 7 d ranged from 47% (United Kingdom) to 81% (Mexico), and that of sugary drinks ranged from 62% (United Kingdom) to 87% (Mexico). Rates of consumption of diet drinks ranged from 26% (Mexico) to 37% (United Kingdom), whereas alcoholic drink consumption rates ranged from 45% (USA) to 52% (Canada). Respondents in Mexico were more likely to consume SSBs and sugary drinks, and in greater amounts, than those in other countries. Respondents in the United Kingdom were more likely to consume diet drinks than those in Australia, Canada, and Mexico, and greater amounts of diet drinks were consumed in the United Kingdom and the USA. Across countries, younger respondents and males were more likely to consume greater amounts of SSBs and sugary drinks.

**Conclusions:**

Most adult respondents across all countries consumed SSBs and sugary drinks, with greater consumption in Mexico and the USA. Consumption varied greatly across countries, but patterns of association among subpopulations were relatively similar.

## Introduction

Beverages play an important role in energy intake and diet quality and can contribute to positive energy balance. Many beverages are calorically dense and nutrient poor as well as contributing little to satiation (1, 2). High consumption of sugar-sweetened beverages (SSBs) has received particular scrutiny, because consumption is associated with weight gain, diabetes, metabolic syndrome, cardiovascular disease, cancer, dental caries (3–11), and all-cause mortality (12). There is also public health interest in patterns of consumption of alcoholic drinks, and particularly binge or heavy drinking, as they relate to overall caloric intake and subsequent obesity (13). Studies suggest there is little or no compensation for alcoholic beverage energy intake, and high amounts of consumption result in increased food and total energy intakes (14).

International data suggest that beverage sales are highest in North America, followed by Latin America, Australasia, and Western Europe (15). Dietary intake data from national surveys collected at differing time points over the past 15 y are consistent with sales data, showing considerable variation in beverage intake among adults across the globe (16). In 2010, mean global intake of SSBs was 4.6 ounces (135 mL)/d, ranging from 2.8 ounces (80 mL)/d in low-income countries to 5.1 ounces (150 mL)/d in upper-middle-income countries (16). Fruit juice consumption, with a mean of 1.3 ounces (40 mL)/d, varied significantly by region and by country income level (16). Beverage intake also differs between population subgroups. For example, over the past 15 y, global SSB intake was highest among men aged 20–39 y of age and fruit juice intake was highest among women 20–39 y of age (16).

Patterns of beverage intake are changing. Market data suggest stable aggregate sales of nonalcoholic beverages, with considerable shifts occurring among beverage categories (17). In particular, sales of carbonated beverages (particularly caloric varieties) have decreased over the past 6 y, whereas sales of bottled water have increased (17). These trends are supported by the limited evidence available from dietary intake data, which have reported overall declines in sugary drink consumption among the general population (18–21) and children (22). Global trends in alcoholic beverages are less consistent: some countries such as the United Kingdom and Australia have seen decreases in alcoholic drink intakes (22, 23), whereas others such as the USA have seen increases over the past several decades (24).

In response to changes in purchasing and intake patterns, the beverage industry is increasingly diversifying the beverage market and adjusting marketing strategies to maintain or increase global sales (25). In particular, the proportion of artificially sweetened beverages (diet and low-calorie varieties) and “hybrid” beverages sweetened from both caloric and noncaloric sources is increasing (26–29). Although there is some evidence that intake of diet beverages with only low-calorie sweeteners decreased in North America from 2000 to 2014 (15), more recent evidence on how intake patterns are changing is lacking.

Currently, few studies directly compare beverage intake between countries. Most international comparative studies have used sales and purchasing data, which do not provide accurate information on differential consumption within populations (15). Further, data comparing beverage intake across countries are scarce and often combine data collected using different dietary assessment methods (16). Lastly, dietary intake data are typically collected using 24-h food recalls, which may not capture intake of episodically consumed beverages, such as energy drinks and alcohol (16).

The aims of the current study were to examine intake of commonly consumed beverages among adults across 5 upper-middle- and high-income countries, to compare the frequency of consumption and amounts consumed of commonly consumed beverages between countries, and to examine patterns of consumption among population subgroups.

## Methods

Data for adults aged ≥18 y and residing in Australia, Canada, Mexico, the United Kingdom, and the USA were drawn from the 2017 wave of the International Food Policy Study (IFPS), and supplemented with data from the 2017 wave of the Canada Food Study (CFS), as detailed below. The IFPS sample was recruited from the Nielsen Consumer Insights Global Panel, which maintains and/or has partner panels in each country (https://www.nielsen.com/us/en/about-us/panels/). The panels are recruited using both probability and nonprobability sampling methods, with a standardized recruitment sampling strategy used across countries in November/December 2017.

Individuals were eligible to participate in the IFPS if they were 18 y of age or older and resided in the country of the panel in which they were participating. For the IFPS, email invitations with a unique link to the online screener were sent to a random sample of panelists who met the inclusion criteria. After screening, eligible potential respondents were provided with information about the study and asked to provide consent. Consenting respondents who completed the survey received remuneration in accordance with their panel's usual incentive structure. Quota sampling was conducted with quotas for age and sex, and efforts were made to target low-education panelists, according to national census estimates. Further details on the IFPS are available elsewhere (30). The CFS sample (ages 18–32 y) was recruited from a pool of Canadian participants formerly recruited in person to participate in an online panel of young adults from 5 Canadian cities. CFS respondents were sent a unique survey link to complete the online survey, which used survey items that were the same as, or comparable with, those included in the IFPS.

For a participant flowchart, see **Supplemental Figure 1**. A total of 25,692 adults completed the online IFPS survey, which examined a wide variety of aspects related to dietary patterns, nutrition, and health. Sample sizes were determined for measurements in the overall study to examine changes in cross-sectional data over time. Surveys were conducted in English in Australia and the United Kingdom; Spanish in Mexico; English or French in Canada; and English or Spanish in the USA. A data integrity check was included part way through the survey, whereby participants were asked to select the current month from a list. Those providing an invalid response or who failed to state their sex at birth or region were removed from the analytic sample (*n* = 6814, 26.5%), leaving 18,878 IFPS respondents. Comparable data for 979 respondents were drawn from the CFS in Canada only (31). The current analyses were further restricted to individuals with complete data on beverage intake, consisting of 16,397 adults (82.6% of the entire IFPS/CFS sample) (Australia, *n* = 3264; Canada, *n* = 2745; Mexico, *n* = 3152; the United Kingdom, *n* = 3221; and the USA, *n* = 4015). The studies received ethics clearance through a University of Waterloo Research Ethics Committee (ORE# 21460 for the IFPS and ORE# 30893 for the CFS).

### Beverage intake measures

Beverage intake was examined using the Beverage Frequency Questionnaire (BFQ) (32), adapted for each country. A version of the BFQ has been examined relative to 7-d food records among young adults aged 16–30 y in Canada, showing moderate to high correlation and agreement between estimates of frequency of consumption and total volume of each of the beverage categories consumed for 14 of 17 beverage categories (between 0.46 and 0.91 for the number of drinks, 0.48 and 0.95 for volume) and lower correlation for 3 of 17 categories (coffee with cream or sugar, specialty coffee, and hard alcohol with no mix) (32). The BFQ had moderate to good correlations for aggregate categories of SSBs (0.53 for the number of drinks and 0.54 for volume), sugary drinks (0.63 for the number of drinks and 0.55 for volume), alcoholic beverages (0.58 for the number of drinks and 0.78 for volume), and all drinks (0.62 for the number of drinks and 0.59 for volume) (32). The BFQ used in the current study was modified from the original BFQ to examine frequency of intake of 22 beverage categories over the past 7 d. Categories were based on commonly and episodically consumed beverages. Participants were first asked, “During the PAST 7 DAYS, HOW MANY DRINKS did you have in each category below?” for 18 nonalcoholic beverage categories and 4 alcoholic beverage categories, with examples of beverages to prompt recognition. For each beverage category selected, participants were shown an array of common beverage containers (between 2 and 7 options, as well as “less” and “more” options) of varying sizes and shapes and were asked, “For each type of drink, what size did you USUALLY have? If you had different sizes, select the picture that is closest to the average size.” The BFQ was tailored in each country to provide product examples and typical beverage container sizes commonly sold in each market. The BFQ beverage categories for each of the countries and an example of the size images used can be found in **Supplemental Table 1** and **Supplemental Figure 2**, respectively.

### Beverage intake analysis

Two definitions of sweetened beverages were applied. SSBs refers to beverages with considerable amounts of *added sugars*, defined as sugars and syrups added to foods or beverages during processing and preparation. The SSB definition included regular soda, sweetened fruit drinks, regular (i.e., caloric) flavored water, sports drinks, energy drinks, chocolate milk/other flavored milk, and sweetened smoothies/protein shakes/drinkable yogurt. “Sugary drinks” included beverages containing *free sugars*, defined as mono- and disaccharides added to foods or beverages by the manufacturer, cook, or the consumer, as well as the sugars naturally present in honey, syrups, and 100% fruit juice (11). Fruit juice has often been excluded from definitions of SSBs, reflecting beliefs that the nutritional contribution of 100% juice is greater than that of soda or fruit drinks (33); however, 100% juice is a major source of sugar, contributes to energy intake (15), and has been linked to several health concerns (11). The term “sugary drinks” in this study thus refers to SSBs plus 100% fruit or vegetable juice.

Total volume for each beverage category of interest was calculated by multiplying the number of drinks consumed in the previous 7 d by the usual serving size selected for that category, as per the BFQ methods (32). If participants selected “more” than the largest container, the container volume was coded as 125% of the largest container listed (e.g., if the largest container was 500 mL, the volume was coded as 625 mL). Similarly, if the participant selected “less” than the smallest container, the volume was coded as 50% of the smallest container listed (e.g., if the smallest container was 250 mL, the volume was coded as 125 mL). If participants indicated they had consumed a beverage category but selected “don't know” for the container size, they were assigned the container size most commonly reported for that category within their country. Responses between 70 and 100 drinks/wk for any single beverage category (i.e., >10 drinks from that category per day, not including water) were recoded to 70 drinks/d. Responses >100 drinks/wk in any single beverage category were excluded because these were characterized as nonsensical/implausible data, and outliers, defined as total volume of beverages reported >36 L [∼2 times the IQR in the highest country (United Kingdom) considering multiple years of data], were recoded to missing and all beverage data were excluded, as per recommendations for dealing with outlier dietary data in the US National Cancer Institute's Dietary Assessment Primer (34).

### Sociodemographics

Sociodemographic characteristics were assessed as part of the larger IFPS survey using country-specific survey measures adopted from population-level surveys within each country (35–40). The resulting data were recoded and harmonized for comparison across countries. Sociodemographic variables included age (continuous), sex at birth, ethnicity (majority group or minority group), education (low, medium, or high), a subjective measure of perceived income adequacy, and BMI calculated using self-reported height and weight and categorized according to the WHO classification (41). Education level was categorized as “low” (i.e., completed secondary school or less), “medium” (i.e., some postsecondary qualifications), or “high” (i.e., university degree or higher) according to country-specific criteria related to the highest level of formal education attained. For perceived income adequacy, participants were asked, “Thinking about your total monthly income, how difficult or easy is it for you to make ends meet?” (very difficult, difficult, neither easy nor difficult, easy, very easy, reclassified as difficult/very difficult and neither easy nor difficult/easy/very easy).

### Statistical analyses

Analyses were conducted using SAS version 9.4 (SAS Institute). Data were weighted using poststratification sample weights constructed based on population estimates from the census in each country based on age group, sex, and region (42–46). Estimates reported are weighted.

Descriptive statistics examined the frequency of consumption of beverage categories and types in each of the countries. Means and percentiles were used to describe the distribution of intakes among the overall sample and those who reported that they consumed the beverage in each country. Because the beverage intake data were severely positively skewed, attempts were made to apply log and Box–Cox transformations to normalize the distribution of residuals. Owing to the large proportion of 0s (i.e., nonconsumers of a given beverage category), adequate transformation was not achieved, barring the use of a single regression model to examine correlates of intake among consumers and nonconsumers combined. Thus, for each beverage category, separate logistic regression models were conducted to examine the likelihood of any consumption of the beverage category relative to correlates, and linear regression models were conducted to examine correlates of the amount consumed among those who reported consuming the beverage type. Amounts consumed were log transformed for inclusion in linear regression models. Given the nonlinear relation, results from models were back-transformed and converted to percentage change (increase or decrease) in the volume consumed to assist interpretation. All models were tested to ensure that assumptions of linear regression modeling were met (data not shown).

All models adjusted for the country variable and the aforementioned sociodemographic variables of age (continuous), sex at birth, ethnicity, education, perceived income adequacy, and BMI, which were selected a priori as important contributors to dietary intake and known to differ between the sample populations in each country. Individuals with missing data for any of the sociodemographic variables were removed on a case-wise basis from each model. Separate regression models were used to estimate differences in the associations between sociodemographic correlates and consumption between countries using interaction terms. Multiplicative interactions between country and each sociodemographic variable were entered individually into the base model and interactions that were significant at a level of *P* < 0.05 were then entered into the base model simultaneously. Interactions that were not significant at *P* < 0.01 from the multi-interaction model were removed, leaving only significant interactions in the final interaction model.

For all regressions, survey-aware procedures were used to account for finite sampling methods and 99% CIs are reported for adjusted odds ratios (AORs), unless otherwise noted.

## Results

**Table 1** describes characteristics of the overall sample and within each country.

**TABLE 1:** Demographic characteristics for respondents included in the sample (weighted)^1^

	Overall (*n =* 16,397)	Australia (*n* = 3264)	Canada (*n* = 2745)	Mexico (*n* = 3152)	United Kingdom (*n* = 3221)	USA (*n* = 4015)
Age, y	40.3 ± 13.4	40.9 ± 13.4	39.9 ± 13.8	37.5 ± 12.4	41.4 ± 13.3	41.3 ± 13.6
Sex						
Female	8193 (50.0)	1658 (50.8)	1364 (49.7)	1603 (50.9)	1559 (48.4)	2009 (50.0)
Ethnicity^2^						
Majority group	12,829 (78.2)	2696 (82.6)	1822 (66.4)	2719 (86.3)	2877 (89.3)	2714 (67.6)
Minority group	3375 (20.6)	543 (16.7)	863 (31.4)	394 (12.5)	308 (9.6)	1267 (31.5)
Not stated	193 (1.2)	20 (0.8)	60 (2.2)	37 (1.2)	36 (1.1)	34 (0.8)
Education^3^						
Low	3291 (20.1)	883 (26.7)	368 (13.4)	465 (15.1)	784 (24.3)	792 (19.7)
Medium	4122 (25.1)	1151 (35.2)	928 (33.8)	353 (11.4)	927 (28.8)	758 (18.9)
High	8876 (54.1)	1217 (37.3)	1430 (52.1)	2231 (72.5)	1488 (46.2)	2452 (61.1)
Not stated	108 (0.7)	23 (0.7)	24 (0.7)	27 (0.9)	23 (0.7)	13 (0.3)
Perceived income adequacy^4^						
Very difficult/difficult	4322 (26.4)	828 (25.4)	636 (23.2)	1296 (41.1)	814 (25.2)	747 (18.6)
Neither easy nor difficult/easy/very easy	11,826 (72.1)	2397 (73.4)	2022 (73.6)	1820 (57.7)	2369 (73.6)	3218 (80.2)
Not stated	249 (1.5)	39 (1.2)	87 (3.2)	36 (1.2)	38 (1.2)	50 (1.2)
BMI, kg/m^2^						
Underweight (<18.5)	473 (2.9)	89 (2.7)	86 (3.1)	78 (2.5)	109 (3.4)	111 (2.8)
Normal weight (18.5–24.9)	6129 (37.4)	1174 (36.0)	1140 (41.5)	1276 (40.5)	1076 (33.4)	1463 (36.4)
Overweight (25.0–29.9)	4601 (28.0)	843 (25.8)	740 (27.0)	1056 (33.5)	718 (22.3)	1245 (31.0)
Obesity (≥30.0)	2922 (17.8)	643 (19.7)	458 (16.7)	552 (17.5)	393 (12.2)	875 (21.8)
Not stated	2272 (13.9)	515 (15.8)	321 (11.7)	189 (6.0)	925 (28.7)	321 (8.0)

1 *n* = 16,397. Values are means ± SDs or *n* (%).

2 Ethnic categories in each country as per census questions asked in each country: *1*) Australia, majority = only speaks English at home, minority = speaks a language besides English at home; *2*) Canada, majority = white, minority = other ethnicity; *3*) Mexico, majority = nonindigenous, minority = indigenous; *4*) United Kingdom, majority = white, minority = other ethnicity; *5*) USA, majority = white, minority = other ethnicity.

3 Education level was categorized as “low” (i.e., completed secondary school or less), “medium” (i.e., some postsecondary qualifications), or “high” (i.e., university degree or higher) according to country-specific criteria related to the highest level of formal education attained.

4 Participants were asked, “Thinking about your total monthly income, how difficult or easy is it for you to make ends meet?” (very difficult, difficult, neither easy nor difficult, easy, or very easy, reclassified as difficult/very difficult and neither easy nor difficult/easy/very easy).

### Rates of consumption of beverage categories

**FIGURE 1:**
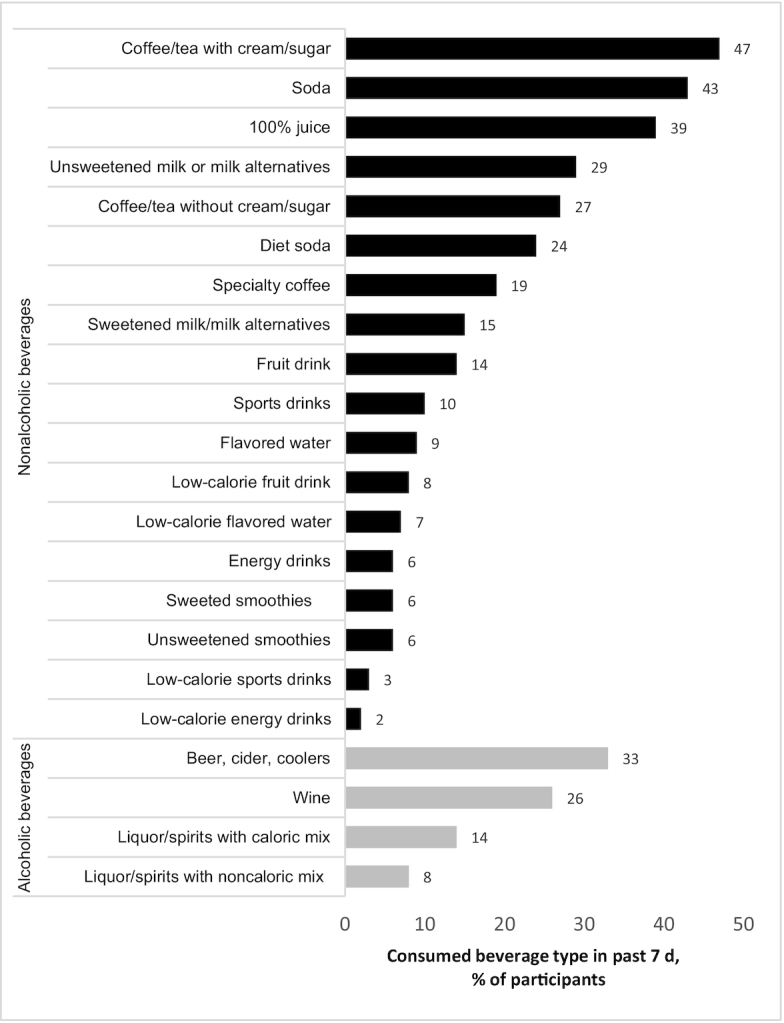
Unadjusted percentage of the study population who reported consuming each beverage category at least once in the past 7 d in Australia, Canada, Mexico, the United Kingdom, and the USA (all countries) (*n* = 16,397).

Respondents reported consuming a mean ± SD of 3.9 ± 2.8 beverage categories in the past 7 d, with the mean ranging from 3.4 in Australia to 5.1 in Mexico. In the full sample (all countries), coffee and tea with cream or sugar was the most commonly consumed category, followed by regular (caloric) soda and 100% fruit or vegetable juice (**Figure 1**). The beer, cider, and alcoholic coolers (prepared, sweetened alcoholic beverages) category was the fourth most commonly consumed beverage category, and the most prevalent of all alcoholic beverages, followed by wine. **Supplemental Figure 3** shows the rates of consumption in each category by country. There were significant differences in likelihood of consumption between countries for all beverage categories, except for low-calorie energy drinks, after adjusting for sociodemographic covariates (*P* < 0.01 for all, data not shown).

**FIGURE 2:**
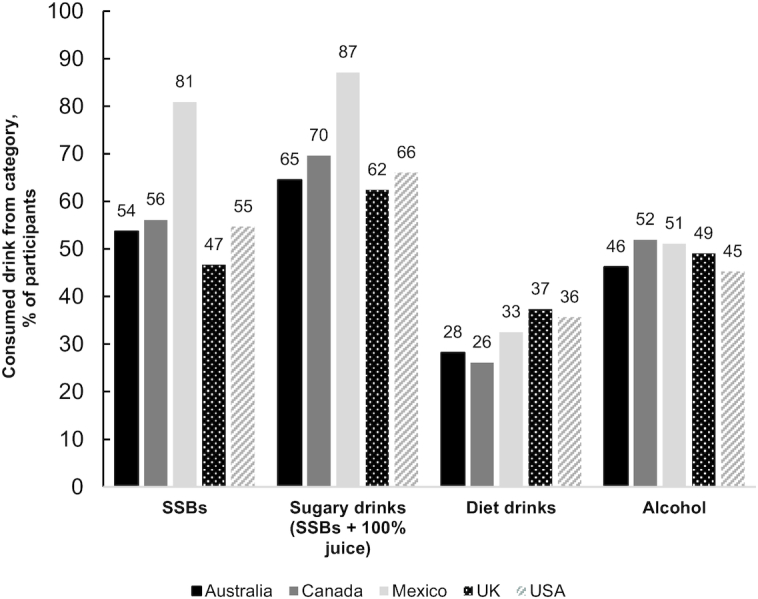
Unadjusted percentage of the study population that consumed any SSBs, sugary drinks (SSBs and 100% juice), diet drinks, and alcohol in the past 7 d by country (*n* = 16,397). SSB, sugar-sweetened beverage.

**Figure 2** shows the percentages of the study population who reported consuming SSBs, sugary drinks, diet drinks, and alcoholic drinks in the past 7 d, by country. Differences in the likelihood of consumption of all of the beverage types by country were observed (**Table 2**).

**TABLE 2:** AORs for any reported consumption of any SSBs, sugary drinks (SSBs + 100% juice), diet drinks, and alcoholic drinks in the past 7 d across countries^1^

	SSBs	Sugary drinks	Diet drinks	Alcoholic drinks
Country				
Australia vs. Canada	0.91 (0.77, 1.07)	0.82 (0.69, 0.97)	1.07 (0.90, 1.28)	0.79 (0.67, 0.92)
Australia vs. Mexico	0.26 (0.22, 0.32)	0.28 (0.22, 0.35)	0.82 (0.69, 0.98)	0.89 (0.76, 1.05)
Australia vs. United Kingdom	1.29 (1.10, 1.51)	1.08 (0.91, 1.27)	0.66 (0.56, 0.78)	0.91 (0.78, 1.07)
Australia vs. USA	0.90 (0.77, 1.05)	0.91 (0.78, 1.07)	0.72 (0.62, 0.85)	1.10 (0.94, 1.28)
Canada vs. Mexico	0.29 (0.24, 0.36)	0.34 (0.27, 0.43)	0.77 (0.64, 0.92)	1.13 (0.96, 1.35)
Canada vs. United Kingdom	1.42 (1.20, 1.68)	1.32 (1.11, 1.58)	0.61 (0.52, 0.73)	1.16 (0.98, 1.38)
Canada vs. USA	0.99 (0.84, 1.16)	1.12 (0.95, 1.33)	0.68 (0.57, 0.80)	1.40 (1.19, 1.64)
Mexico vs. United Kingdom	4.87 (4.01, 5.91)	3.87 (3.12, 4.80)	0.80 (0.67, 0.95)	1.03 (0.87, 1.21)
Mexico vs. USA	3.39 (2.81, 4.09)	3.28 (2.66, 4.06)	0.88 (0.75, 1.04)	1.23 (1.05, 1.45)
United Kingdom vs. USA	0.70 (0.59, 0.82)	0.85 (0.72, 1.00)	1.10 (0.93, 1.29)	1.20 (1.02, 1.41)
Age	0.97 (0.96, 0.97)	0.98 (0.97, 0.98)	1.00 (1.00, 1.01)	1.02 (1.01, 1.02)
Sex				
Female vs. male	0.73 (0.65, 0.81)	0.76 (0.68, 0.85)	0.98 (0.88, 1.09)	0.75 (0.68, 0.83)
Ethnicity^2^				
Majority vs. minority group	0.83 (0.72, 0.96)	0.87 (0.75, 1.01)	1.25 (1.08, 1.44)	1.65 (1.44, 1.90)
Education^3^				
High vs. low	0.78 (0.68, 0.90)	0.92 (0.79, 1.07)	1.26 (1.10, 1.46)	1.60 (1.40, 1.83)
High vs. medium	0.76 (0.66, 0.86)	0.86 (0.75, 0.98)	1.20 (1.05, 1.36)	1.30 (1.15, 1.47)
Low vs. medium	0.97 (0.83, 1.13)	0.93 (0.79, 1.10)	0.95 (0.81, 1.11)	0.81 (0.70, 0.94)
Perceived income adequacy^4^				
Easy vs. difficult	0.82 (0.73, 0.93)	0.88 (0.77, 1.01)	1.19 (1.06, 1.35)	1.26 (1.13, 1.42)
BMI				
Underweight vs. normal weight	1.07 (0.77, 1.48)	1.21 (0.84, 1.73)	0.94 (0.67, 1.31)	0.77 (0.56, 1.07)
Underweight vs. overweight	0.95 (0.68, 1.32)	1.10 (0.76, 1.59)	0.76 (0.54, 1.07)	0.78 (0.56, 1.08)
Underweight vs. obesity	0.83 (0.59, 1.17)	1.04 (0.71, 1.51)	0.56 (0.40, 0.80)	1.06 (0.76, 1.49)
Underweight vs. missing	0.95 (0.67, 1.35)	1.18 (0.81, 1.73)	0.79 (0.55, 1.13)	1.26 (0.89, 1.78)
Normal weight vs. overweight	0.89 (0.78, 1.01)	0.91 (0.79, 1.05)	0.81 (0.71, 0.92)	1.01 (0.89, 1.14)
Normal weight vs. obesity	0.78 (0.67, 0.90)	0.86 (0.73, 1.01)	0.61 (0.52, 0.70)	1.37 (1.19, 1.59)
Normal weight vs. missing	0.89 (0.75, 1.06)	0.98 (0.82, 1.17)	0.84 (0.71, 1.00)	1.63 (1.38, 1.92)
Overweight vs. obesity	0.88 (0.75, 1.02)	0.94 (0.80, 1.11)	0.75 (0.64, 0.87)	1.37 (1.18, 1.59)
Overweight vs. missing	1.00 (0.84, 1.21)	1.07 (0.89, 1.30)	1.04 (0.87, 1.25)	1.62 (1.36, 1.93)
Obesity vs. missing	1.15 (0.94, 1.40)	1.14 (0.93, 1.40)	1.40 (1.15, 1.69)	1.19 (0.98, 1.43)

1 *n* = 15,919. Values are AORs (99% CIs). AOR, adjusted odds ratio; SSB, sugar-sweetened beverage.

2 Ethnic categories in each country as per census questions asked in each country: *1*) Australia, majority = only speaks English at home, minority = speaks a language besides English at home; *2*) Canada, majority = white, minority = other ethnicity; *3*) Mexico, majority = nonindigenous, minority = indigenous; *4*) United Kingdom, majority = white, minority = other ethnicity; *5*) USA, majority = white, minority = other ethnicity.

3 Education level was categorized as “low” (i.e., completed secondary school or less), “medium” (i.e., some postsecondary qualifications), or “high” (i.e., university degree or higher) according to country-specific criteria related to the highest level of formal education attained.

4 Participants were asked, “Thinking about your total monthly income, how difficult or easy is it for you to make ends meet?” [very difficult, difficult, neither easy nor difficult, easy, or very easy, reclassified as DIFFICULT (difficult/very difficult) and EASY (neither easy nor difficult/easy/very easy)].

Mexico had the greatest percentage of respondents who reported consuming any SSBs and the United Kingdom had the smallest percentage. In adjusted models, all between-country contrasts for consumption of any SSBs were significant, except between Australia, Canada, and the USA. The likelihood of consuming any SSBs was higher among younger respondents, males, those with low and medium education compared with high education, those in minority ethnicity groups, those for whom it was difficult to make ends meet, and those with obesity compared with those with normal weight.

The between-country contrasts for any sugary drink consumption (SSBs + 100% juice) were slightly different from those observed for SSBs, such that for sugary drinks, there were differences between Canada and each of Australia and the United Kingdom but no differences in the likelihood of consuming any sugary drinks between Australia, the United Kingdom, and the USA, or between Canada and the USA. There were also fewer sociodemographic differences observed in consumption of sugary drinks: younger participants and males were more likely than their counterparts to report consuming any sugary drinks (Table 2).

Overall, consumption of any diet drinks was most common in the United Kingdom and least common in Canada. All between-country differences were significant except differences between Australia and Canada, and the USA and each of Mexico and the United Kingdom. Compared with the findings for SSBs, dissimilar patterns of sociodemographic differences emerged: those in the majority ethnicity group, those with high education compared with medium and low education, and those for whom it was easy to make ends meet were more likely to consume diet drinks. Similar to the patterns for SSBs, those with obesity compared with underweight, normal BMI, overweight, or missing data and those with overweight compared with normal BMI were more likely to consume diet drinks.

Consumption of alcohol was most common in Canada and least common in the USA. Respondents in Canada were more likely to report consuming alcohol than those in Australia and the USA, and Mexico and UK respondents were more likely to report alcohol consumption than those in the USA. All sociodemographic variables were significantly associated with consuming alcoholic beverages: older participants, males, those in the majority ethnicity group, those who found it easier to make ends meet, those with higher education, and those with normal weight or overweight compared with those with obesity or missing height and weight data were more likely to consume alcoholic beverages.

### Amount of beverages consumed among the entire sample and those who consumed each category

**Table 3** shows the overall means ± SEs for the amounts consumed of SSBs, sugary drinks, diet drinks, and alcoholic drinks among the entire sample (including consumers and nonconsumers) and among consumers only. Table 3 also shows the median and the 10th, 25th, 75th, and 90th percentiles for the amounts consumed among consumers of the respective drinks. **Supplemental Table 2** shows the mean, median, and percentiles among the entire population, **Supplemental Table 3** shows the frequency of consuming any SSBs or sugary drinks among population subgroups, **Supplemental Table 4** shows the mean amount consumed (including consumers and nonconsumers) among various population subgroups by country for SSBs and sugary drinks, and **Supplemental Table 5** shows the same for diet drinks and alcoholic drinks.

**TABLE 3:** Overall means, medians, and percentiles for volume consumed of SSBs, sugary drinks, diet drinks, and alcohol in the past 7 d among the entire sample and consumers of each beverage type^1^

		Entire sample in past 7 d, mL	Consumers in past 7 d, mL					
	Country	Mean ± SE	Mean ± SE	10th percentile	25th percentile	Median	75th percentile	90th percentile
SSBs	Australia	1380 ± 59	2560 ± 98	350	600	1240	2850	5990
	Canada	1280 ± 56	2280 ± 90	249	580	1340	2630	5340
	Mexico	3020 ± 76	3730 ± 86	495	1180	2530	4790	8650
	United Kingdom	1250 ± 55	2690 ± 100	315	628	1320	3230	6630
	USA	1650 ± 62	3010 ± 100	341	667	1660	3910	7410
Sugary drinks	Australia	1790 ± 67	2770 ± 96	363	736	1570	3100	6240
(SSBs + 100% juice)	Canada	1780 ± 62	2560 ± 81	343	717	1590	3000	5830
	Mexico	3710 ± 84	4260 ± 90	592	1490	3000	5520	9450
	United Kingdom	1820 ± 65	2920 ± 93	359	745	1740	3490	6980
	USA	2080 ± 69	3150 ± 94	351	710	1760	4090	7430
Diet drinks	Australia	738 ± 44	2610 ± 130	287	710	1430	2970	5720
	Canada	731 ± 49	2800 ± 160	336	681	1260	2970	6830
	Mexico	852 ± 48	2620 ± 120	347	618	1560	3410	6200
	United Kingdom	1260 ± 66	3370 ± 150	320	713	1920	3980	7340
	USA	1310 ± 55	3690 ± 150	350	833	2250	4610	8400
Alcohol	Australia	1140 ± 52	2470 ± 100	297	655	1420	3000	5320
	Canada	1080 ± 46	2080 ± 77	286	531	1210	2570	4760
	Mexico	940 ± 44	1840 ± 78	334	541	1150	2160	4100
	United Kingdom	1220 ± 51	3490 ± 88	329	659	1500	3030	5750
	USA	868 ± 34	1920 ± 63	283	533	1190	2370	4290

1 SSB, sugar-sweetened beverage.

**Supplemental Figures 4–6** show the contribution of each of the beverage categories to total SSB consumption, diet drink consumption, and alcohol consumption among the total sample. The greatest contributor to the volume of SSBs was soda in all countries, and the smallest contributors were sweetened smoothies (Australia, United Kingdom) and energy drinks (Canada, Mexico, USA). In all countries, the greatest contributor to diet drinks was diet soda (ranging from 50% to 73% of volume, depending on the country) and the greatest contributor to alcoholic drinks was beer, cider, and coolers (ranging from 54% to 62% of volume, depending on the country), with little contribution from spirits/liquor (e.g., rum, vodka, gin) with no or a noncaloric mix.

### Amount of SSBs, sugary drinks, diet drinks, and alcohol consumed among consumers

Based on linear regression analyses (**Table 4**), among those who reported consuming SSBs in the past 7 d (*n* = 9271), the amount consumed was greater in Mexico than in all other countries, and greater in the USA than in Australia, Canada, and the United Kingdom. There was also a small difference between Canada and the United Kingdom. All sociodemographic factors examined, with the exception of ethnicity, were associated with the amount of SSB intake. Younger participants, males, those with low and medium levels of education compared with high, those with obesity compared with those with underweight, normal weight, or overweight and to some extent, those for whom it was difficult to make ends meet consumed greater amounts of SSBs. In a separate model that tested interactions between country and each of the sociodemographic variables, there was a significant interaction between country and education (*P* = 0.004). Education was not associated with SSB intake in Mexico, but was in all other countries (data not shown).

**TABLE 4:** Linear regression coefficients for the volume of SSBs, sugary drinks (SSBs + 100% juice), diet drinks, and alcoholic drinks consumed in the past 7 d among consumers relative to country and sociodemographic characteristics^1^

	SSBs (*n* = 9271)	Sugary drinks (SSBs + 100% juice) (*n* = 11,097)	Diet drinks (*n* = 5171)	Alcoholic drinks (*n* = 7765)
Country				
Australia vs. Canada	4 (−8, 16)	−1 (−10, 10)	−1 (−15, 15)	11 (−2, 25)
Australia vs. Mexico	−46 (−51, −40)	−47 (−52, −42)	−9 (−22, 7)	19 (5, 35)
Australia vs. United Kingdom	−9 (−19, 3)	−9 (−18, 0.3)	−20 (−31, −8)	−4 (−15, 9)
Australia vs. USA	−20 (−28, −10)	−14 (−22, −5)	−29 (−38, −18)	12 (−0.2, 26)
Canada vs. Mexico	−48 (−53, −42)	−47 (−52, −42)	−8 (−22, 9)	8 (−5, 22)
Canada vs. United Kingdom	−12 (−22, −0.3)	−9 (−18, 1)	−19 (−31, −5)	−13 (−23, −2)
Canada vs. USA	−23 (−31, −13)	−14 (−21, −5)	−28 (−37, −16)	2 (−9, 14)
Mexico vs. United Kingdom	69 (51, 89)	73 (56, 90)	−12 (−25, 2)	−19 (−28, −9)
Mexico vs. USA	48 (33, 64)	63 (49, 79)	−22 (−33, −9)	−6 (−16, 6)
United Kingdom vs. USA	−12 (−22, −1)	−5 (−15, 5)	−11 (−22, −3)	17 (3, 31)
Age	−0.8 (−1, −0.6)	−0.7 (−0.9, −0.5)	0.7 (0.3, 1)	−0.05 (−0.2, 0.3)
Sex (female vs. male)	−22 (−27, −17)	−23 (−28, −18)	−11 (−19, −2)	−38 (−43, −33)
Ethnicity^2^ (majority vs. minority group)	−2 (−11, 6)	−7 (−14, −0.3)	17 (4, 33)	29 (16, 44)
Education^3^				
High vs. low	−23 (−29, −16)	−21 (−27, −14)	2 (−10, 17)	−6 (−16, 5)
High vs. medium	−21 (−28, −14)	−21 (−26, −14)	−4 (−15, 8)	−11 (−19, −3)
Low vs. medium	2 (−8, 13)	0.0 (−9, 9)	−7 (−19, 8)	−5 (−16, 7)
Perceived income adequacy^4^ (easy vs. difficult)	−8 (−15, −1)	−6 (−13, 0.3)	−2 (−12, 10)	3 (−6, 13)
BMI				
Underweight vs. normal weight	−11 (−27, 8)	−7 (−22, 10)	−25 (−44, 0.1)	−1 (−23, 27)
Underweight vs. overweight	−16 (−31, 3)	−10 (−25, 7)	−33 (−50, −10)	−11 (−31, 14)
Underweight vs. obesity	−27 (−41, −10)	−19 (−33, −3)	−46 (−60, −27)	−8 (−29, 20)
Underweight vs. missing	−20 (−35, −0.5)	−15 (−29, 3)	−42 (−57, −22)	0.5 (−24, 31)
Normal weight vs. overweight	−6 (−13, 3)	−4 (−10, 4)	−10 (−20, 1)	−10 (−18, −2)
Normal weight vs. obesity	−18 (−25, −9)	−13 (−20, −5)	−28 (−37, −18)	−7 (−17, 4)
Normal weight vs. missing	−10 (−19, 1)	−8 (−17, 1)	−23 (−34, −10)	1 (−13, 16)
Overweight vs. obesity	−13 (−21, −3)	−10 (−18, −1)	−20 (−29, −9)	4 (−7, 16)
Overweight vs. missing	−4 (−15, 8)	−5 (−15, 6)	−14 (−28, 1)	12 (−3, 30)
Obesity vs. missing	10 (−4, 25)	5 (−4, 18)	7 (−10, 27)	8 (−7, 26)

1 Values are % Δ volumes (99% CIs). SSB, sugar-sweetened beverage; % Δ, difference in the volume between comparison groups (calculated for back-transformed βs for interpretation because all volumes consumed were log-transformed for analysis).

2 Ethnic categories in each country as per census questions asked in each country: *1*) Australia, majority = only speaks English at home, minority = speaks a language besides English at home; *2*) Canada, majority = white, minority = other ethnicity; *3*) Mexico, majority = nonindigenous, minority = indigenous; *4*) United Kingdom, majority = white, minority = other ethnicity; *5*) USA, majority = white, minority = other ethnicity.

3 Education level was categorized as “low” (i.e., completed secondary school or less), “medium” (i.e., some postsecondary qualifications), or “high” (i.e., university degree or higher) according to country-specific criteria related to the highest level of formal education attained.

4 Participants were asked, “Thinking about your total monthly income, how difficult or easy is it for you to make ends meet?” [very difficult, difficult, neither easy nor difficult, easy, very easy, reclassified as DIFFICULT (difficult/very difficult) and EASY (neither easy nor difficult/easy/very easy)].

The associations between sociodemographic characteristics and amounts of sugary drinks (SSBs + 100% juice) reported among consumers of this beverage category (*n* = 11,097) were similar to those observed for SSBs, with greater influence of ethnicity and slightly lesser difference by perceived income adequacy. Country differences were also similar, although there was a smaller magnitude in the difference between the United Kingdom and each of Canada and the USA in the sugary drinks model. In a separate model that tested interactions between country and sociodemographic variables, there was a significant interaction between BMI and country, whereby there was no relation between BMI and sugary drink intake in Canada, Mexico, and the United Kingdom, but there were significant associations in Australia and the USA (data not shown).

Compared with SSBs and sugary beverages, there were fewer significant between-country differences in the amounts of diet drinks reported among consumers of this category (*n* = 5171). Consumers in the United Kingdom and the USA reported a higher volume of diet drinks than those in Australia and Canada, and US consumers reported a higher volume than consumers in Mexico. Males, those in the majority ethnicity group, and older consumers consumed a greater volume of diet drinks than their counterparts. There were significant differences in intake across all BMI categories except when comparing those with a normal BMI to those who with underweight or overweight BMI. In a separate model, there was a significant interaction between country and perceived income adequacy (*P* = 0.004, data not shown), such that there were between-country differences in the amounts of diet drinks consumed among those for whom it was easy to make ends meet, with lower consumption in Australia compared to Mexico (−20%, *P*= 0.002), the United Kingdom (−20%, *P* = 0.0004) and the USA (−32%, *P* < 0.0001), lower consumption in Canada compared to Mexico (−17%, *P*= 0.008), the United Kingdom (−18%, *P*= 0.003) and the USA (−31%, *P*< 0.0001), lower consumption in Mexico compared to the USA (−16%, *P*= 0.009), and lower consumption in the United Kingdom compared to the USA (−15%, *P*= 0.007). There were fewer between-country differences among those for whom it was more difficult to make ends meet, with the only difference observed between respondents in Mexico and the United Kingdom (−33% lower consumption in Mexico, *P* < 0.0001).

Among those who consumed alcoholic drinks (*n* = 7765), respondents in the United Kingdom consumed more than those in Canada, Mexico, and the USA, and respondents in Australia consumed more than respondents in Mexico. Males, those in the majority ethnicity group, and those with a high level of education compared with those with a medium level of education consumed greater amounts of alcoholic beverages. In a separate model, there was a significant interaction between age and country (*P* = 0.005), such that there was a steeper age gradient in Australia compared to the difference by age in Canada and the USA and a steeper gradient in the UK compared to the difference by age in the USA, and an interaction between ethnicity and country (*P* = 0.006), such that ethnicity was significant in Australia, Canada, and the USA, but not in Mexico or the United Kingdom (data not shown).

## Discussion

Overall, almost two-thirds of a sample of adults in 5 upper-middle- and high-income countries consumed SSBs in the last week and three-quarters consumed sugary drinks. The prevalence of consumption and volume consumed of SSBs and sugary drinks were substantial across countries, with prevalence and amounts consumed highest in Mexico, followed by the USA, and lower in Canada, the United Kingdom, and Australia. Diet beverages were consumed by one-quarter to one-third of each sample, whereas approximately half of all participants consumed alcoholic drinks. This study demonstrates important variations in beverage consumption across countries.

Estimates of beverage consumption from the BFQ observed in this study demonstrated similar patterns to those documented based on national dietary intake data and sales data from each of the countries. The patterns of data were similar to estimates from the Global Burden of Disease (GBD) study (16) and an analysis of sales data (15), although differences between the USA and other countries were smaller in the current study. The current findings are consistent with research indicating very high amounts of sugary beverage consumption in Mexico (15, 47).

Consumption of 100% juice was common in all countries and highest in Canada. These findings differ from those of the GBD study, which estimated lower amounts of fruit juice consumed across most countries than the amounts of consumption documented here (16). The difference may be due to older data, and may reflect trends of increased consumption of fruit juices due to substitution away from SSBs (16).

The correlates of SSB intake identified in this study are consistent with the broader literature indicating that SSB consumption is higher among males, younger age groups, those of lower socioeconomic status, and those of minority ethnicities (48–50). Interestingly, the patterns of sugary drink consumption were somewhat distinct from patterns of SSB consumption. Sugary drinks (e.g., including 100% juice) were just as likely to be reported by high-income and high-education participants as by those on the lower end of the socioeconomic spectrum. There were few notable between-country differences in the sociodemographic patterns of consumption of drinks with sugar with the exception of the relationship between the amount of SSB consumption and education in Mexico and between the amount of sugary drink consumption and BMI in several countries. These results serve as a reminder that defining the issue of sugar intake from beverages as a problem of people with low socioeconomic status is not justified, and that public health efforts are needed at the population level.

This study identified significant between-country differences in diet drink intake, which may relate to differences in social norms and preferences for these types of beverages, the type and availability of diet drinks in each country's market, as well as perceptions of low-calorie sweeteners within countries (51, 52). This study also found a strong relation between having obesity and greater consumption of diet drinks. Although nonnutritive sweeteners have been recognized as safe for regulatory purposes (53), their impact on health is still debated and the role of diet drinks in weight maintenance or weight gain is unclear (54–57). Substantial consumption of diet beverages in all of the countries, and between-country differences, deserve attention because these may shift over time.

Approximately half of the sample reported consuming alcoholic drinks in the past 7 d. Although this study found greater differences in intake of alcoholic beverages by age in several country comparisons, the proportion of the sample that consumed alcohol was substantial among all age groups. Few studies have comprehensively examined beverage intake including both alcoholic and nonalcoholic categories, despite the important caloric contribution of alcohol to population-level energy intake (14).

### Strengths and limitations

This study represents a multicountry comparison using a relatively large sample size, with consistent methods used across countries. Examining both alcoholic and nonalcoholic categories is a considerable strength of this study, because modeling research has suggested that changes to purchasing and intake patterns among nonalcoholic beverages could influence alcoholic beverage patterns (58). Respondents were recruited using nonprobability-based sampling; therefore, the findings do not provide nationally representative estimates. For example, although the data were weighted by age, sex, and region, the Mexico sample had a higher average level of education than that based on census estimates (44), whereas the proportion of respondents who reported a BMI that would be categorized as having obesity was somewhat lower than national estimates in each of the 5 countries (59). Because of differences in the types of specialty coffees available and consumed between countries and the relative amounts of sugars in these coffees, these were excluded from the definition of SSBs and sugary drinks used in this study, resulting in underestimation of the prevalence and amounts of consumption that may vary across countries. In addition, data collection was conducted in November/December, which is summer in the Southern Hemisphere (Australia) but autumn or early winter in all other study countries. This may have resulted in increased intake of both nonalcoholic and alcoholic drinks in Australia, because beverage intake is likely to be higher in the warmer summer season (60–62). Although adjustment for seasonality was not possible in the current study, future work should consider whether or not there is a seasonality effect that may differ between countries.

All self-reported frequency questions, including the BFQ, are subject to measurement error. In addition, patterns of intake for alcohol in particular may not be consistent each week, and thus may not indicate “typical” intake patterns. As noted, body weight status is a predictor of misreporting of dietary intake, as is education. To the extent that the samples across the 5 countries differed with respect to these characteristics, the between-country comparisons may be affected. Further, given the rampant and growing weight stigma, examination of trends over time in beverage intake in relation to body weight should be approached with an abundance of caution. The changing beverage industry, with increasing beverage options that are “hybrids” of both caloric and noncaloric sweeteners, may also create challenges for consumers in accurately reporting the beverages they consumed and may contribute to reporting errors for 100% juice, fruit drinks, and diet drinks, which may be challenging to distinguish. However, this source of measurement error would be expected to be similar across countries. Lastly, given the brevity of the tool, not all beverage types may be captured in the categories listed (e.g., nonalcoholic beer beverages).

### Policy implications and future research

A number of regulatory approaches have been introduced to modify beverage intake patterns, including taxes on beverages with excess added or free sugars (63), restricting the marketing of less healthy beverages to children (64–66), point-of-purchase (67, 68) and mass media (69–71) educational interventions to increase awareness of the health effects of sugary drink consumption, and changes to dietary guidance around beverages (72). This comparison may serve as an important baseline for future work to evaluate alcoholic and nonalcoholic policy interventions that are implemented in the forthcoming years: although this study is not able to assess the impact of policies in place before 2017, it is well positioned to examine future changes as governments in IFPS countries implement new approaches to address sugar intake. The results have implications for several potential policy interventions as results from the IFPS are tracked over time. For example, evidence suggests that consumers may perceive 100% juice as a healthier alternative to SSBs and dietary guidance in many countries continues to consider a small glass of juice as a serving of vegetable and fruit (72–74). As a result, 100% juice is often excluded from policies aimed at reducing caloric beverage intake, despite containing similar amounts of calories and sugars due to its high free sugars content (75, 76) and associations between higher rates of fruit juice intake and type 2 diabetes (7, 77) and coronary heart disease (78). Therefore, future research may examine the extent to which policies implemented to reduce SSB intake influence 100% fruit juice consumption, and differences between policies that include and exclude 100% fruit juice in definitions of beverages to avoid or limit.

With regards to diet drinks, how policy interventions such as sugary drink taxes and efforts to reformulate sugar influence the intake of nonnutritive sweeteners deserves greater attention as trends in intake of these beverages continue to shift. Lastly, current policies aimed at sugary drink reduction seldom acknowledge intake of alcoholic beverages, and beverage-related policies could have unintended consequences on alcoholic drink intake (58). Trends in alcohol consumption should be tracked over time to examine the impact of nonalcoholic beverage policies on alcohol use.

### Conclusion

In conclusion, the findings of this study suggest important differences in amounts of beverage consumption across these 5 countries, with mostly similar sociodemographic correlates of consumption within countries. Longitudinal comparisons of beverage intake across countries, using future waves of the IFPS study, will be important to shed light on the impacts of national-level policies.

## Supplementary Material

nxaa324_Supplemental_File

## Notes

The first 2 waves of the International Food Policy Study were funded by a population health intervention research operating grant from the Canadian Institutes of Health Research (CIHR). Additional support was provided by a CIHR–Public Health Agency of Canada Applied Public Health Chair (to DH). JA receives funding from the Centre for Diet and Activity Research, a UK Clinical Research Collaboration (UKCRC) Public Health Research Centre of Excellence. Funding from the British Heart Foundation, Cancer Research UK, Economic and Social Research Council, Medical Research Council, the National Institute for Health Research, and the Wellcome Trust, under the auspices of the UKCRC, is gratefully acknowledged. This work was supported by the Medical Research Council MC_UU_12015/6. GS is supported by National Heart Foundation of Australia Heart Foundation Future Leader Fellowship 102035.

Author disclosures: David Hammond has served as a paid expert witness on behalf of public health authorities in response to litigation from the food and beverage industry. All the other authors report no conflicts of interest.

Supplemental Tables 1–5 and Supplemental Figures 1–6 are available from the “Supplementary data” link in the online posting of the article and from the same link in the online table of contents at https://academic.oup.com/jn/.

Data Availability: Data described in the article, code book, and analytic code will be made available upon request pending application and approval by DH.

Abbreviations used: BFQ, Beverage Frequency Questionnaire; CFS, Canada Food Study; GBD, Global Burden of Disease; IFPS, International Food Policy Study; SSB, sugar-sweetened beverage.
