# Supplementary material for: Nonalcoholic and Alcoholic Beverage Intakes by Adults across 5 Upper-Middle- and High-Income Countries
Source: J Nutr. 2020 Nov 26;151(1):140–51. doi: 10.1093/jn/nxaa324 (PMC7779239; doi:10.1093/jn/nxaa324)
Supplement: nxaa324_Supplemental_File [file nxaa324_supplemental_file.docx]

**Supplemental Table 1**. Beverage categories included in the Beverage Frequency Questionnaire (BFQ) for Australia, Canada, Mexico, United Kingdom and United States

| **AUSTRALIA**  For example, if you had 2 regular soft drinks during the past 7 days, you would enter 2 in that box.  If you had 1 regular soft drink EACH day, you would enter 7 in that box.  **# OF DRINKS**  **Regular soft drink** (Coke, Pepsi, Fanta, Sprite, ginger beer, etc.) ****Not including diet soft drink***  **Diet soft drink** (Pepsi Max, Diet Coke, etc.)  **100% fruit or vegetable juice** (orange juice, apple juice, etc.)  **Sweetened fruit drinks** (Prima, fruit punch/cocktail, iced tea, etc.)  **Low-/no-calorie fruit drinks** (diet lemonade, unsweetened iced tea, etc.)  **Tap water**  **Plain bottled water**  **Regular flavoured waters or vitamin waters with calories** (cordial, vitamin water, Pump flavoured water)  **Low-/no-calorie** **flavoured waters or vitamin waters** (diet cordial, LQD+, Cottees Squirtz, etc.)  **Regular sports drinks** (Gatorade, Powerade, etc.)  **Low-/no-calorie** **sports drinks** (G2, Powerade Zero, etc.)  **Regular energy drinks** (Red Bull, V, Mother, etc.)  **Low-/no-calorie** **energy drinks** (Red Bull Sugarfree, etc.)  **Dairy milk** or unsweetened milk alternatives (unsweetened soy, almond, etc.) as a beverage ****NOT including milk consumed in cereal, etc****.*  **Flavoured milk**, incl. chocolate milk, iced coffee and hot chocolate, or sweetened milk alternatives (sweetened soy, almond, etc.)  **Coffee or tea**, **with milk/cream or sugar**  **Coffee or tea**, **no milk/cream or sugar,** with or without artificial sweetener  **Espresso style coffees with milk** (lattes, mochas, frappucinos, macchiatos, etc.)  **Sweetened smoothies, protein shakes, or drinkable yogurt**  **Unsweetened smoothies, protein shakes, or drinkable yogurt**  **Beer, cider, alcopop**  **Wine** (red, white or sparkling)  **Spirits with mixer, cocktails that have calories/sugar** (rum & coke, gin & tonic, margarita, martini, etc.)  **Spirits with no mixer or diet/non-caloric mix** (shots, whiskey on the rocks, vodka & soda, rum & diet, etc.) |
| --- |
| **CANADA**  **During the PAST 7 DAYS, HOW MANY DRINKS did you have in each category below?**  For example, if you had 2 regular sodas or pops during the past 7 days, you would enter 2 in that box. ​​​​​​​If you had 1 regular soda or pop EACH day, you would enter 7 in that box.  **# OF DRINKS**  **Regular soda or pop** (Coke, Pepsi, 7-up, Sprite, root beer, etc) ****Not including diet pop***  **Diet soda or pop** (Diet Pepsi, Coke Zero, etc.)  **100% fruit or vegetable juice** (orange juice, apple juice, etc.)  **Sweetened fruit drinks** (lemonade, iced tea, SunnyD, fruit punch/cocktail, etc.)  **Low-/no-calorie** **fruit drinks** (diet lemonade, unsweetened iced tea, etc.)  **Regular flavoured waters or vitamin waters with calories**  **Low-/no-calorie** **flavoured waters or vitamin waters** (Crystal Light, Mio, etc.)  **Regular sports drinks** (Gatorade, Powerade, etc.)  **Low-/no-calorie** **sports drinks** (G2, Powerade Zero, etc.)  **Regular energy drinks** (Red Bull, Rockstar, Monster, etc.)  **Low-/no-calorie** **energy drinks** (Red Bull Sugarfree, etc.)  **White milk** or unsweetened milk alternatives (unsweetened soy, almond, etc.) as a beverage ****NOT including milk consumed in cereal, etc****.*  **Chocolate or flavoured milk** (incl. hot chocolate), or sweetened milk alternatives (sweetened soy, almond, etc.)  **Coffee or tea, with milk/cream or sugar**  **Coffee or tea, no milk/cream or sugar,** with or without artificial sweetener  **Speciality coffees** (lattes, mochas, frappucinos, macchiatos, etc.)  **Sweetened smoothies, protein shakes, or drinkable yogurt**  **Unsweetened smoothies, protein shakes, or drinkable yogurt**  **Beer, cider, coolers**  **Wine** (red or white)  **Hard alcohol with mix, cocktails that have calories** (rum & coke, gin & tonic, margarita, caesar, etc.)  **Hard alcohol with no mix or non-caloric mix** (shots, whiskey on the rocks, vodka & soda, rum & diet, etc.)  None of the above  Don’t know  Refuse to answer |
| **MEXICO**  **En los ÚLTIMOS 7 DÍAS, ¿CUÁNTAS BEBIDAS tomó de cada una de las siguientes categorías?**  Por ejemplo, si bebió 2 refrescos normales durante los últimos 7 días, escriba “2” en esa casilla. Si tomó 1 refresco normal CADA día, escriba “7” en esa casilla.  [PROGRAMMER NOTE: Responses must be numeric and between 0-100; only allow participant to select 1 of none of the above, DK or R]  **# DE BEBIDAS**  **Bebida refrescos normal** (Coca-cola, Pepsi, 7-Up, Sprite, cerveza de raíz, etc.) ****No incluya bebidas refrescos de dieta***  **Bebidas refrescos de dieta** (Pepsi de dieta, Coca-cola Zero, etc.)  **Jugo 100% de fruta o de verduras** (jugo de naranja, de manzana, etc.)  **Bebidas de fruta endulzadas** (limonada, té helado, SunnyD, ponche/coctel de frutas, etc.)  **Bebidas de fruta sin/bajas en calorías** (limonada de dieta, té helado sin azúcar, etc.)  **Agua de la llave (hervida o sin hervir) o de garrafón**  **Agua simple/natural embotellada**  **Aguas de sabor o vitaminadas normales con calorías**  **Aguas de sabor o vitaminadas sin/bajas en calorías** (Crystal Light, Mio, etc.)  **Bebidas deportivas** (Gatorade, Powerade, etc.)  **Bebidas deportivas sin/bajas en calorías** (G2, Powerade Zero, etc.)  **Bebidas energéticas normales** (Red Bull, Rockstar, Monster, etc.)  **Bebidas energéticas sin/bajas en calorías** (Red Bull Sugarfree, etc.)  **Leche de vaca** o sustitutos de leche sin endulzar (de soya, almendra sin endulzar, etc.), consumidas como bebida ****NO incluya leche consumida con cereal, etc****.*  **Leche con chocolate** **o algún otro sabor** (incluido chocolate caliente) o alternativas a la leche sin endulzar (de soya, almendra endulzada, etc.)  **Café o té, con leche/crema o azúcar**  **Café o té, sin leche/crema ni azúcar,** con o sin endulzante artificial  **Cafés de especialidad** (lattes, mochas, frappucinos, macchiatos, etc.)  **Smoothies, licuados de proteína o yogurt para beber con azúcar añadida**  **Smoothies, licuados de proteína o yogurt para beber sin azúcar añadida**  **Cerveza, sidra, bebidas a base de vino (coolers)**  **Vino** (tinto o blanco)  **Bebidas alcohólicas mezcladas, cocteles con calorías** (ron con Coca-cola, gin & tonic, coctel Margarita, etc.)  **Bebidas alcohólicas no mezcladas o con mezclas no calóricas** (shots, whiskey en las rocas, vodka con soda, ron con Coca-cola dietetica, etc.) |
| **UNITED KINGDOM**  **During the PAST 7 DAYS, HOW MANY DRINKS did you have in each category below?**  For example, if you had 2 regular fizzy drinks during the past 7 days, you would enter 2 in that box.  If you had 1 regular fizzy drink EACH day, you would enter 7 in that box.  [PROGRAMMER NOTE: Responses must be numeric and between 0-100; only allow participant to select 1 of none of the above, DK or R]  **# OF DRINKS**  **Fizzy drinks** (Coke, Pepsi, 7-up, Sprite, root beer, etc) ****Not including diet fizzy drinks***  **Diet fizzy drinks** (Diet Pepsi, Coke Zero, etc.)  **100% fruit or vegetable juice** (orange juice, apple juice, etc.)  **Sweetened fruit drinks** (lemonade, iced tea, SunnyD, fruit punch/cocktail, etc.)  **Low-/no-calorie** **fruit drinks** (diet lemonade, unsweetened iced tea, etc.)  **Tap water**  **Plain bottled water**  **Sweetened flavoured waters or vitamin waters with calories**  **Low-/no-calorie** **flavoured waters or vitamin waters** (Touch of Fruit, Perfectly Clear, etc.)  **Regular sports drinks** (Lucozade Sport, Powerade, etc.)  **Low-/no-calorie** **sports drinks** (Lucozade Sport Low Cal, Powerade Zero, etc.)  **Regular energy drinks** (Red Bull, Rockstar, Monster, etc.)  **Low-/no-calorie** **energy drinks** (Red Bull Sugarfree, etc.)  **White milk** or unsweetened milk alternatives (unsweetened soy, almond, etc.) as a beverage ****NOT including milk consumed in cereal, etc****.*  **Chocolate or flavoured milk** (incl. hot chocolate), or sweetened milk alternatives (sweetened soy, almond, etc.)  **Coffee or tea, with milk/cream or sugar**  **Coffee or tea, no milk/cream or sugar,** with or without artificial sweetener  **Speciality coffees** (lattes, mochas, frappucinos, macchiatos, etc.)  **Sweetened smoothies, protein shakes, or drinkable yogurt**  **Unsweetened smoothies, protein shakes, or drinkable yogurt**  **Beer, cider, lager**  **Wine** (red, white or rose)  **Spirits with mixers, cocktails that have calories** (rum & coke, gin & tonic, margarita, etc.)  **Spirits with no mixers or non-caloric mix** (shots, whiskey on the rocks, vodka & soda, rum & diet coke, etc.) |
| **UNITED STATES**  **During the PAST 7 DAYS, HOW MANY DRINKS did you have in each category below?**  For example, if you had 2 regular sodas or pops during the past 7 days, you would enter 2 in that box. ​​​​​​​If you had 1 regular soda or pop EACH day, you would enter 7 in that box.  [PROGRAMMER NOTE: Responses must be numeric and between 0-100; only allow participant to select 1 of none of the above, DK or R]  **# OF DRINKS**  **Regular soda or pop** (Coke, Pepsi, 7-up, Sprite, root beer, etc) ****Not including diet pop***  **Diet soda or pop** (Diet Pepsi, Coke Zero, etc.)  **100% fruit or vegetable juice** (orange juice, apple juice, etc.)  **Sweetened fruit drinks** (lemonade, iced tea, SunnyD, fruit punch/cocktail, etc.)  **Low-/no-calorie** **fruit drinks** (diet lemonade, unsweetened iced tea, etc.)  **Tap water**  **Plain bottled water**  **Regular flavoured waters or vitamin waters with calories**  **Low-/no-calorie** **flavoured waters or vitamin waters** (Crystal Light, Mio, etc.)  **Regular sports drinks** (Gatorade, Powerade, etc.)  **Low-/no-calorie** **sports drinks** (G2, Powerade Zero, etc.)  **Regular energy drinks** (Red Bull, Rockstar, Monster, etc.)  **Low-/no-calorie** **energy drinks** (Red Bull Sugarfree, etc.)  **White milk** or unsweetened milk alternatives (unsweetened soy, almond, etc.) as a beverage ****NOT including milk consumed in cereal, etc****.*  **Chocolate or flavored milk** (incl. hot chocolate), or sweetened milk alternatives (sweetened soy, almond, etc.)  **Coffee or tea, with milk/cream or sugar**  **Coffee or tea, no milk/cream or sugar,** with or without artificial sweetener  **Speciality coffees** (lattes, mochas, frappucinos, macchiatos, etc.)  **Sweetened smoothies, protein shakes, or drinkable yogurt**  **Unsweetened smoothies, protein shakes, or drinkable yogurt**  **Beer, cider, coolers**  **Wine** (red or white)  **Hard alcohol with mix, cocktails that have calories** (rum & coke, gin & tonic, margarita, etc.)  **Hard alcohol with no mix or non-caloric mix** (shots, whiskey on the rocks, vodka & soda, rum & diet, etc.) |

**Supplemental Figure 1. Participant Flow Chart: IFPS 2017**

979 adults ages 18-30 from the Canada Food Study

*n=*6,814 from IFPS sample removed for poor data quality (failed to state sex at birth or region OR provided invalid response to data integrity question)

*n=*3,460 did not have complete beverage intake data (missing frequency and/or volume for at least one beverage category or implausible data)

25,692 adults completed International Food Policy Study online survey from 5 countries

18,878 adults in IFPS sample

16,397 adults in analytical sample

19,857 adults in complete sample

**Supplemental Figure 2. Samples images from the Canadian BFQ used to portray commonly consumed beverage serving sizes**


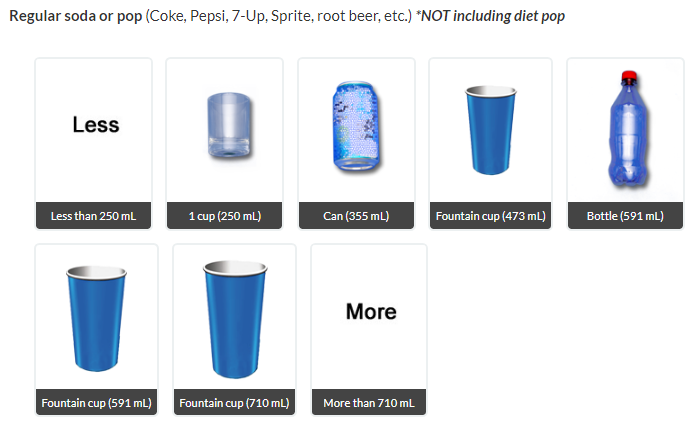


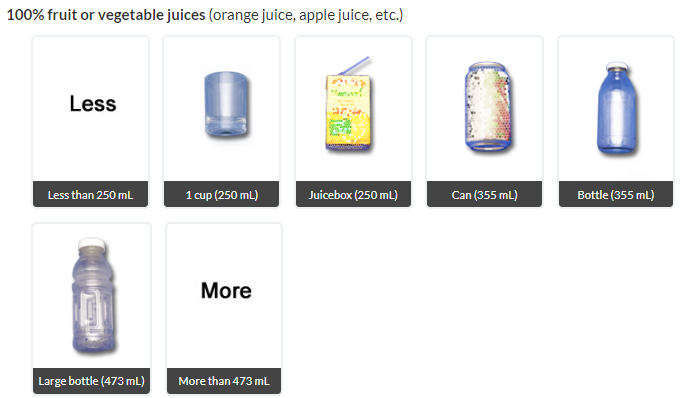


*(Additional information and images available upon request from authors)*

**Supplementary Figure 3**. The percentage of the study population that consumed at least one beverage from each drink category by country (*N*=16,397)

**Supplemental Table 2.** Overall mean, median and percentiles of consumption of SSBs, sugary drinks, diet drinks and alcohol in the past 7 days among the entire sample (*N=*16,397)

|  | **Country** | **Mean (SE) (mL)** | **10^th^ %ile** | **25^th^ %ile** | **Median (mL)** | **75^th^ %ile** | **90^th^ %ile** |
| --- | --- | --- | --- | --- | --- | --- | --- |
| **SSBs** | Australia | 1380 (59) | 0 | 0 | 243 | 1490 | 3730 |
|  | Canada | 1280 (56) | 0 | 0 | 328 | 1470 | 3490 |
|  | Mexico | 3020 (76) | 0 | 476 | 1770 | 4140 | 7640 |
|  | UK | 1250 (55) | 0 | 0 | 0 | 1240 | 3550 |
|  | US | 1650 (62) | 0 | 0 | 332 | 1770 | 4950 |
| **Sugary drinks**  **(SSBs + 100% juice)** | Australia | 1790 (67) | 0 | 0 | 726 | 2000 | 4620 |
|  | Canada | 1780 (62) | 0 | 0 | 839 | 2250 | 4730 |
|  | Mexico | 3710 984) | 0 | 886 | 2480 | 5000 | 8860 |
|  | UK | 1820 (65) | 0 | 0 | 630 | 2090 | 4980 |
|  | US | 2080 (69) | 0 | 0 | 709 | 2480 | 5800 |
| **Diet drinks** | Australia | 738 (44) | 0 | 0 | 0 | 307 | 2250 |
|  | Canada | 731 (49) | 0 | 0 | 0 | 190 | 1970 |
|  | Mexico | 852 (48) | 0 | 0 | 0 | 586 | 2740 |
|  | UK | 1260 (66) | 0 | 0 | 0 | 992 | 3540 |
|  | US | 1310 (55) | 0 | 0 | 0 | 1060 | 4100 |
| **Alcohol** | Australia | 1140 (52) | 0 | 0 | 0 | 1250 | 3430 |
|  | Canada | 1080 (46) | 0 | 0 | 131 | 1320 | 3080 |
|  | Mexico | 940 (44) | 0 | 0 | 126 | 1130 | 2520 |
|  | UK | 1220 (51) | 0 | 0 | 0 | 1490 | 3590 |
|  | US | 868 (34) | 0 | 0 | 0 | 1060 | 2600 |

**Supplemental Table 3.** Frequency of consuming SSBs, sugary drinks, diet drinks and alcoholic drinks across socio-demographic subgroups stratified by country (%) (*N=*16,397)

|  | **Australia**  (*n=*3,264) | | | | **Canada**  (*n=*2,745) | | | | **Mexico**  (*n=*3,152) | | | | **United Kingdom**  (*n=*3,221) | | | | **USA**  (*n=*4,015) | | | |
| --- | --- | --- | --- | --- | --- | --- | --- | --- | --- | --- | --- | --- | --- | --- | --- | --- | --- | --- | --- | --- |
|  | **SSBs** | **Sugary drinks** | **Diet**  **drinks** | **Alcohol** | **SSBs** | **Sugary drinks** | **Diet**  **drinks** | **Alcohol** | **SSBs** | **Sugary drinks** | **Diet**  **drinks** | **Alcohol** | **SSBs** | **Sugary drinks** | **Diet**  **drinks** | **Alcohol** | **SSBs** | **Sugary drinks** | **Diet**  **drinks** | **Alcohol** |
| **Sex**  Female  Male | 49  58 | 61  68 | 27  30 | 42  50 | 52  60 | 67  72 | 26  26 | 50  54 | 79  83 | 86  89 | 30  36 | 45  57 | 45  48 | 61  64 | 38  37 | 44  53 | 51  59 | 62  70 | 35  36 | 43  48 |
| **Ethnicity^1^**  Majority group  Minority group | 54  55 | 65  64 | 28  27 | 48  35 | 54  61 | 69  72 | 27  24 | 58  41 | 80  85 | 86  92 | 33  30 | 52  45 | 45  58 | 61  74 | 38  34 | 51  30 | 51  64 | 63  70 | 39  28 | 49  36 |
| **Education^2^**  Low  Medium  High | 57  56  49 | 66  67  62 | 30  26  29 | 41  47  49 | 63  55  55 | 74  66  71 | 27  26  26 | 49  52  52 | 85  83  80 | 88  89  87 | 23  27  35 | 39  42  55 | 43  54  44 | 56  67  63 | 35  38  38 | 40  47  55 | 59  58  52 | 68  69  64 | 30  34  38 | 34  37  51 |
| **Perceived Income Adequacy^3^**  Very difficult/Difficult  Neither easy nor difficult/easy/very easy | 59  52 | 67  64 | 27  29 | 41  48 | 59  55 | 72  69 | 25  27 | 46  54 | 82  80 | 87  88 | 29  35 | 47  55 | 49  46 | 64  62 | 36  38 | 41  52 | 67  52 | 74  64 | 29  37 | 37  48 |
| **BMI, kg/m^2^**  Underweight (<18.5)  Normal weight (18.5-24.9)  Overweight (25.0-29.9)  Obesity (≥30.0)  Not stated | 55  53  53  56  55 | 71  64  63  66  64 | 23  25  28  35  28 | 36  49  52  44  35 | 58  55  58  55  56 | 78  70  72  65  66 | 14  21  27  40  26 | 41  56  54  47  42 | 86  81  81  81  78 | 91  88  87  86  84 | 22  30  35  35  31 | 33  52  56  48  36 | 55  46  42  45  50 | 68  63  60  60  64 | 36  34  37  49  36 | 43  53  58  45  40 | 59  52  53  59  58 | 67  63  66  70  66 | 33  31  38  41  33 | 36  45  50  44  33 |
| **Age^4^, y**  18-29  30-39  40-49  50-64 | 69  59  53  40 | 76  66  63  56 | 31  31  25  27 | 35  44  44  58 | 67  56  53  49 | 77  69  65  66 | 20  27  31  28 | 52  49  48  57 | 88  85  79  69 | 92  90  85  79 | 26  32  36  38 | 39  54  54  60 | 64  55  44  32 | 76  68  60  52 | 36  42  38  34 | 38  41  47  62 | 65  64  54  42 | 73  72  65  58 | 27  36  32  43 | 39  44  45  50 |
| ^1^ Ethnic categories in each country as per census questions asked in each country: 1) Australia majority=only speaks English at home, minority=speaks a language besides English at home; 2) Canada majority=White, minority=other ethnicity; 3) Mexico majority=Non-indigenous, minority=indigenous; 4) United Kingdom majority=White, minority=other ethnicity; 5) US majority=White, minority=other ethnicity.  ^2^ Education level was categorized as ‘low’ (i.e. completed secondary school or less), ‘medium’ (i.e. some post-secondary qualifications), or ‘high’ (i.e. university degree or higher) according to country-specific criteria related to the highest level of formal education attained.  ^3^ Participants were asked “Thinking about your total monthly income, how difficult or easy is it for you to make ends meet?” (very difficult, difficult, neither easy nor difficult, easy, very easy, reclassified as DIFFICULT (difficult/very difficult) and EASY (neither easy nor difficult/easy/very easy).  ^4^Age is reported as a categorical variable for demonstration purposes, but is modelled as a continuous variable in all models reported within the manuscript. | | | | | | | | | | | | | | | | | | | | |

**Supplemental Table 4.** Mean consumption of SSBS and sugary drinks (SSBs + 100% juice) among the entire sample across socio-demographic groups by country (mL) (*N=*16,397)

|  | **Australia**  (*n=*3,264) | | | **Canada**  (*n=*2,745) | | **Mexico**  (*n=*3,152) | | | **United Kingdom**  (*n=*3,221) | | | **USA**  (*n=*4,015) | |
| --- | --- | --- | --- | --- | --- | --- | --- | --- | --- | --- | --- | --- | --- |
|  | **SSBs**  **Mean (SE)**  **mL** | **Sugary drinks Mean (SE)**  **mL** | **SSBs**  **Mean (SE)**  **mL** | | **Sugary drinks Mean (SE)**  **mL** | **SSBs**  **Mean (SE)**  **mL** | **Sugary drinks Mean (SE)**  **mL** | **SSBs**  **Mean (SE)**  **mL** | | **Sugary drinks Mean (SE)**  **mL** | **SSBs**  **Mean (SE)**  **mL** | | **Sugary drinks Mean (SE)**  **mL** |
| **Sex**  Female  Male | 1130 (70)  1630 (95) | 1460 (74)  2130 (110) | 1040 (66)  1510 (90) | | 1470 (74)  2090 (100) | 2670 (110)  3380 (110) | 3320 (120)  4120 (120) | 1190 (77)  1310 (78) | | 1750 (96)  1890 (89) | 1450 (82)  1840 (94) | | 1820 (89)  2334 (100) |
| **Ethnicity^1^**  Majority group  Minority group | 1370 (63)  1390 (150) | 1770 (70)  1860 (200) | 1260 (67)  1310 (110) | | 1790 (74)  1750 (120) | 2590 (80)  3470 (250) | 3640 (89)  4220 (270) | 1220 (58)  1640 (200) | | 1760 (68)  2440 (240) | 1490 (74)  1901 (120) | | 1870 (80)  2534 (130) |
| **Education^2^**  Low  Medium  High | 1600 (120)  1550 (110)  1060 (76) | 1960 (130)  1980 (120)  1490 (100) | 1780 (140)  1430 (120)  1050 (62) | | 2370 (160)  1960 (130)  1520 (68) | 3600 (210)  3280 (235)  2861 (87) | 4210 (230)  3960 (260)  3580 (98) | 1350 (120)  1540 (110)  1030 (72) | | 1800 (140)  2190 (140)  1620 (83) | 2070 (170)  2150 (180)  1360 (65) | | 2460 (180)  2700 (200)  1770 (73) |
| **Perceived Income Adequacy^3^**  Very difficult/Difficult  Neither easy nor difficult/easy/very easy | 1690 (140)  1270 (64) | 2130 (170)  1670 (71) | 1560 (140)  1180 (61) | | 2050 (150)  1700 (68) | 3100 (120)  2990 (98) | 3690 (130)  3765 (110) | 1500 (130)  1170 (60) | | 2000 (140)  1760 (75) | 2350 (189)  1470 (60) | | 2820 (200)  1900 (70) |
| **BMI, kg/m^2^**  Underweight (<18.5)  Normal weight (18.5-24.9)  Overweight (25.0-29.9)  Obesity (≥30.0)  Not stated | 1050 (230)  1290 (94)  1320 (120)  1680 (140)  1350 (140) | 1310 (230)  1660 (100)  1740 (150)  2070 (150)  1890 (180) | 965 (160)  1140 (70)  1410 (140)  1390 (130)  1370 (190) | | 1680 (230)  1590 (77)  2030 (150)  1860 (150)  1820 (201) | 3100 (470)  3050 (120)  2860 (130)  3240 (190)  3050 (350) | 3750 (500)  3850 (140)  3500 (140)  3830 (210)  3670 (380) | 1570 (400)  1140 (85)  1190 (130)  1300 (170)  1380 (95) | | 2370 (450)  1800 (120)  1780 (150)  1630 (190)  1890 (110) | 1600 (420)  1380 (90)  1490 (93)  2030 (150)  2460 (360) | | 1970 (440)  1810 (100)  1890 (100)  2500 (160)  2940 (370) |
| **Age^4^, y**  18-29  30-39  40-49  50-64 | 2020 (130)  1400 (130)  1410 (150)  880 (80) | 2350 (140)  1850 (170)  1830 (170)  1320 (84) | 1510 (91)  1370 (170)  1145 (130)  1090 (72) | | 1960 (100)  1810 (180)  1680 (140)  1670 (85) | 3690 (130)  3150 (140)  2630 (170)  2390 (180) | 4520 (150)  3870 (150)  3230 (180)  3000 (190) | 2130 (150)  1530 (130)  1100 (130)  640 (60) | | 2830 (180)  2050 (140)  1660 (150)  1150 (76) | 2140 (1820)  1900 (120)  1640 (160)  1160 (83) | | 2570 (170)  2340 (130)  2010 (160)  1630 (100) |
| ^1^ Ethnic categories in each country as per census questions asked in each country: 1) Australia majority=only speaks English at home, minority=speaks a language besides English at home; 2) Canada majority=White, minority=other ethnicity; 3) Mexico majority=Non-indigenous, minority=indigenous; 4) United Kingdom majority=White, minority=other ethnicity; 5) US majority=White, minority=other ethnicity.  ^2^ Education level was categorized as ‘low’ (i.e. completed secondary school or less), ‘medium’ (i.e. some post-secondary qualifications), or ‘high’ (i.e. university degree or higher) according to country-specific criteria related to the highest level of formal education attained.  ^3^ Participants were asked “Thinking about your total monthly income, how difficult or easy is it for you to make ends meet?” (very difficult, difficult, neither easy nor difficult, easy, very easy, reclassified as DIFFICULT (difficult/very difficult) and EASY (neither easy nor difficult/easy/very easy).  ^4^Age is reported as a categorical variable for demonstration purposes, but is modelled as a continuous variable in all models reported within the manuscript. | | | | | | | | | | | | | |

**Supplemental Table 5.** Mean consumption of diet drinks and alcoholic drinks among the entire sample across socio-demographic groups by country (mL) (*N=*16,397)

|  | **Australia**  (*n=*3,264) | | **Canada**  (*n=*2,745) | | **Mexico**  (*n=*3,152) | | **United Kingdom**  (*n=*3,221) | | **USA**  (*n=*4,015) | |
| --- | --- | --- | --- | --- | --- | --- | --- | --- | --- | --- |
|  | **Diet drinks**  **Mean (SE)**  **mL** | **Alcoholic drinks Mean (SE)**  **mL** | **Diet drinks**  **Mean (SE)**  **mL** | **Alcoholic drinks Mean (SE)**  **mL** | **Diet drinks**  **Mean (SE)**  **mL** | **Alcoholic drinks Mean (SE)**  **mL** | **Diet drinks**  **Mean (SE)**  **mL** | **Alcoholic drinks Mean (SE)**  **mL** | **Diet drinks**  **Mean (SE)**  **mL** | **Alcoholic drinks Mean (SE)**  **mL** |
| **Sex**  Female  Male | 752 (61)  722 (63) | 811 (56)  1480 (89) | 676 (61)  785 (77) | 794 (46)  1360 (78) | 733 (71)  975 (65) | 639 (64)  1250 (60) | 1310 (95)  1210 (92) | 765 (49)  1650 (85) | 1240 (83)  1390 (95) | 598 (29)  1140 (61) |
| **Ethnicity^1^**  Majority group  Minority group | 783 (51)  517 (67) | 1250 (59)  640 (110) | 847 (68)  479 (57) | 1300 (63)  636 (55) | 866 (52)  819 (130) | 961 (50)  818 (92) | 1290 (72)  1040 (170) | 1300 (56)  522 (84) | 1590 (86)  743 (72) | 1020 (45)  566 (47) |
| **Education^2^**  Low  Medium  High | 830 (89)  699 (75)  713 (67) | 1130 (97)  1380 (100)  931 (69) | 880 (140)  850 (110)  614 (57) | 1200 (130)  1134 (79)  1010 (63) | 522 (97)  718 (160)  946 (58) | 694 (74)  734 (92)  1024 (57) | 1180 (120)  1410 (150)  1214 (91) | 940 (93)  1290 (100)  1340 (74) | 1150 (150)  1340 (150)  1370 (80) | 665 (73)  697 (65)  987 (46) |
| **Perceived Income Adequacy^3^**  Very difficult/Difficult  Neither easy nor difficult/easy/very easy | 853 (96)  700 (49) | 1280 (130)  1090 (54) | 791 (130)  714 (51) | 1010 (93)  1120 (55) | 690 (67)  977 (68) | 777 (48)  1070 (68) | 1350 (140)  1230 (75) | 964 (91)  1320 (61) | 1060 (130)  1380 (70) | 641 (68)  930 (40) |
| **BMI, kg/m^2^**  Underweight (<18.5)  Normal weight (18.5-24.9)  Overweight (25.0-29.9)  Obesity (≥30.0)  Not stated | 514 (180)  437 (38)  812 (97)  1160 (130)  808 (130) | 986 (270)  972 (76)  1360 (110)  1520 (150)  726 (97) | 106 (41)  413 (52)  676 (73)  1689 (200)  782 (160) | 883 (200)  1100 (77)  1190 (87)  1110 (110)  764 (100) | 290 (93)  740 (61)  940 (90)  1060 (150)  757 (160) | 386 (99)  861 (48)  1150 (100)  914 (110)  621 (130) | 768 (210)  1090 (100)  1120 (140)  1940 (220)  1330 (130) | 804 (150)  1204 (82)  1600 (120)  1190 (130)  1010 (97) | 695 (180)  1010 (87)  1390 (110)  1770 (150)  1390 (300) | 562 (140)  808 (56)  1058 (68)  817 (65)  650 (120) |
| **Age^4^, y**  18-29  30-39  40-49  50-64 | 757 (90)  656 (80)  761 (110)  764 (76) | 667 (65)  849 (84)  1300 (170)  1580 (90) | 340 (46)  748 (130)  1010 (150)  895 (80) | 1010 (76)  1020 (130)  876 (85)  1280 (78) | 533 (48)  876 (85)  928 (95)  1160 (150) | 675 (46)  1030 (71)  968 (80)  1150 (150) | 1120 (120)  1470 (140)  1340 (160)  1160 (110) | 735 (61)  977 (101)  1260 (140)  1640 (92) | 675 (73)  1140 (110)  1280 (180)  1880 (120) | 693 (54)  857 (83)  934 (98)  955 (51) |
| ^1^ Ethnic categories in each country as per census questions asked in each country: 1) Australia majority=only speaks English at home, minority=speaks a language besides English at home; 2) Canada majority=White, minority=other ethnicity; 3) Mexico majority=Non-indigenous, minority=indigenous; 4) United Kingdom majority=White, minority=other ethnicity; 5) US majority=White, minority=other ethnicity.  ^2^ Education level was categorized as ‘low’ (i.e. completed secondary school or less), ‘medium’ (i.e. some post-secondary qualifications), or ‘high’ (i.e. university degree or higher) according to country-specific criteria related to the highest level of formal education attained.  ^3^ Participants were asked “Thinking about your total monthly income, how difficult or easy is it for you to make ends meet?” (very difficult, difficult, neither easy nor difficult, easy, very easy, reclassified as DIFFICULT (difficult/very difficult) and EASY (neither easy nor difficult/easy/very easy).  ^4^Age is reported as a categorical variable for demonstration purposes, but is modelled as a continuous variable in all models reported within the manuscript. | | | | | | | | | | |

**Supplemental Figure 4.** Mean SSB consumption in the past 7 days from each of the SSB categories in the overall sample (*N=*16,397)

**Supplementary Figure 5**. Mean diet drink consumption in the past 7 days from each of the diet beverage categories in the overall sample (*N=*16,397)

**Supplementary Figure 6**. Mean alcohol consumption in past 7 days from each of the four alcoholic beverage categories (*N=*16,397)
